# Extraction of tree crowns damaged by *Dendrolimus tabulaeformis* Tsai* et* Liu via spectral-spatial classification using UAV-based hyperspectral images

**DOI:** 10.1186/s13007-020-00678-2

**Published:** 2020-10-09

**Authors:** Ning Zhang, Yueting Wang, Xiaoli Zhang

**Affiliations:** 1 Beijing Key Laboratory of Precision Forestry, Beijing Forestry University, Forestry College, Beijing, 100083 China; 2 Agricultural Information Institute, Chinese Academy of Agricultural Sciences, Beijing, 100081 China; 3 Key Laboratory of Agricultural Big Data, Ministry of Agriculture and Rural Affairs, Beijing, 100081 China

**Keywords:** UAV-based hyperspectral image, Spectral-spatial classification, SVM, EPF, Damaged tree crown extraction

## Abstract

**Background:**

Tree crown extraction is an important research topic in forest resource monitoring. In particular, it is a prerequisite for disease detection and mapping the degree of damage caused by forest pests. Unmanned aerial vehicle (UAV)-based hyperspectral imaging is effective for surveying and monitoring forest health. This article proposes a spectral-spatial classification framework that uses UAV-based hyperspectral images and combines a support vector machine (SVM) with an edge-preserving filter (EPF) for completing classification more finely to automatically extract tree crowns damaged by *Dendrolimus tabulaeformis* Tsai *et* Liu (*D. tabulaeformis*) in Jianping county of Liaoning province, China.

**Results:**

Experiments were conducted using UAV-based hyperspectral images, and the accuracy of the results was assessed using the mean structure similarity index (MSSIM), the overall accuracy (OA), kappa coefficient, and classification accuracy of damaged *Pinus tabulaeformis*. Optimized results showed that the OA of the spectral-spatial classification method can reach 93.17%, and the extraction accuracy of damaged tree crowns is 7.50–9.74% higher than that achieved using the traditional SVM classifier.

**Conclusion:**

This study is one of only a few in which a UAV-based hyperspectral image has been used to extract tree crowns damaged by *D. tabulaeformis*. Moreover, the proposed classification method can effectively extract damaged tree crowns; hence, it can serve as a reference for future studies on both forest health monitoring and larger-scale forest pest and disease assessment.

## Highlights

1. A spectral-spatial method for automatically extracting tree crowns more finely is proposed.
2. Two edge-preserving filter algorithms are used to optimize each SVM classification probability map.
3. RGB images are used as guidance image for edge-preserving filters.
4. Experiments on extracting tree crowns damaged by *D. tabulaeformis* yielded 88.97% accuracy.

## Background

In China, from 2002 to 2013, the total area affected by *Dendrolimus tabulaeformis* Tsai *et* Liu (*D.tabulaeformis*) was 1.5689 × 10^6^ ha, the largest part of which was in Liaoning Province, at, 5.5 × 10^5^ ha [1]. *D.tabulaeformis*, which is the typical disease-causing organism in *Pinus tabuliformis Carrière* (Chinese pine), has contributed toward to great losses [2, 3]. Thus, the effecive detecion of *D.tabulaeformis* are particularly important in coniferous forest healthy surveys. Although traditional field surveys, especially visual inspection, are commonly used for forest pest investigation nowadays, they are not applicable to large forested areas, and has disadvantages of subjective, time-consuming and labor-intensive.

Since the late 1980s, satellite remote sensing technology has been applied to a wide range of pest and disease investigations taking advantage of its multi-spectral and multi-temporal characteristics. Radeloff et al. [4] used pre-outbreak Landsat Thematic Mapper (TM) data to identify factors affecting jack pine budworm population levels as well as peak-outbreak imagery to detect actual defoliation. They were the first to apply spectral mixture analysis to forest damage detection. From then on, Olsson et al. [5], Liang et al. [6] and many other researchers successfully detected forest pests and diseases with high accuracy on a large scale by using different resolution satellite data. However, a large-scale outbreak of *D. tabulaeformis* occurred only one month after the initial occurrence. Hence, satellite sensor images does not fully satisfy the requirements of timeliness and precise monitoring in small-scale and/or heavy-disease areas, because images can be affected by cloud cover, and the spatial resolution is relatively low.

The rapid development of unmanned airborne vehicle (UAV) technology has facilitated low-altitude remote sensing applications. Combined with hyperspectral imaging systems, UAV-based hyperspectral technology offers significant advantages in many fields such as land-use and land-cover studies, agriculture, and power line inspection [7–10]. This technology possesses both the flexibility and the hyperspectral characteristics of UAVs and hyperspectral imaging systems [11]. The advantages of affordability, simple operation, fast imaging speed, and high spatial, spectral, and temporal resolution are indispensable for early detection of *D.tabulaeformis* [12, 13]. UAV-based hyperspectral applications for monitoring forest pests and diseases have witnessed significant development in recent years. Lehmann et al. [14] investigated the utility of UAV-acquired visible-near infrared (VNIR) images to provide reliable remote-sensing data to produce maps of pest infestation levels to support intervention decisions in the management of forests located in northwest Germany. Nasi et al. [15] operated a novel miniaturized hyperspectral frame imaging sensor in the wavelength range of 500–900 nm to identify mature Norwegian spruce trees (*Picea abies L. Karst*.) suffering from infestation, representing a different outbreak phase, by the European spruce bark beetle (*Ips typographus L.*). In the whole process of UAV-based hyperspectral image analysis for forest pest investigation, efficient and accurate tree-crown extraction can provide the most favorable support for the subsequent inversion of damage degree, assessment of pest loss, and mapping of pest-related damage in disease occurrence areas. A leaf-eating pest such as *D. tabulaeformis* can cause 100% defoliation during a severe outbreak. In the case of high crown density or complex understory vegetation conditions, automatic extraction of tree crowns with high precision can effectively avoid the issues of high time consumption and subjective errors involved in artificial plotting the range of the crown.

On the one hand, due to cost, spatial scale and limitations in software, the application of UAV remote sensing technology to forest resource surveys remains in its infancy and the application of hyperspectral imaging technology to forest pest, disease investigation and monitoring remains rare. On the other hand, in current studies on tree crown extraction, light detection and ranging (LiDAR) and high-spatial-resolution images have been widely used as the main data sources with the continuous development of related technologies [16]. The application of UAV-based hyperspectral is relatively few. Thus, this study used UAV-based hyperspectral images as the main data source to extract damaged tree crown information. Many algorithms have been proposed for tree crown extraction in the light of Airborne LiDAR and high-resolution UAV- or satellite-based remote sensing data [17–20]. The commonly used methods include region growing [21], template matching [22], valley following [23], watershed segmentation [24], and 3D modeling [25]. However, the “Hughes” phenomenon often occurs in some of these methods during UAV-based hyperspectral image data analysis, especially when the training data is sparse. This phenomenon leads to a decline in classification accuracy. On this basis, the concept of spectral-spatial classification has been proposed for hyperspectral images to reduce the influence of high dimension on the image classification accuracy [26–28]. With continuous improvement in spatial resolution, the method of spectral-spatial classification has attracted increasing attention and has witnessed steady progress. At present, the spectral-spatial classification framework mainly focuses on two aspects. One is the construction of the spectral-spatial feature extraction algorithm by fusing the image spatial and the spectral information of hyperspectral images, and the other is the construction of the spectral-spatial classification framework to optimize pixel-wise spectral classification results represented by image segmentation and probability optimization.

In this study, by fully considering the characteristics of defoliation caused by *D. tabulaeformis* and the prominent contours of damaged tree crowns, we used UAV-based hyperspectral images and high-definition digital images (RGB) image of *D. tabulaeformis*-damaged Chinese pine forest areas as the main data sources. We propose using a spatial–spectral classification framework to realize automatic extraction of damaged tree crowns. The main targets of this study are as follows:

1. To develop a spectral–spatial classification framework that combines edge-preserving filter (EPF) and support vector machine (SVM) algorithms for UAV-based hyperspectral image classification.
2. To extract *D. tabulaeformis*-damaged tree crowns by using the proposed spectral-spatial classification framework.
3. To analyze the potential of using UAV-based hyperspectral imaging and proposed spectral-spatial classification framework to extract damaged tree crowns.

## Results

### Hyperspectral classification results of SVM

The SVM classification with the G-RBF kernel was used to classify the land cover types of the study area into four categories: bare land, understory vegetation, shadows, and damaged *Pinus tabulaeformis*.

**Fig. 1:**
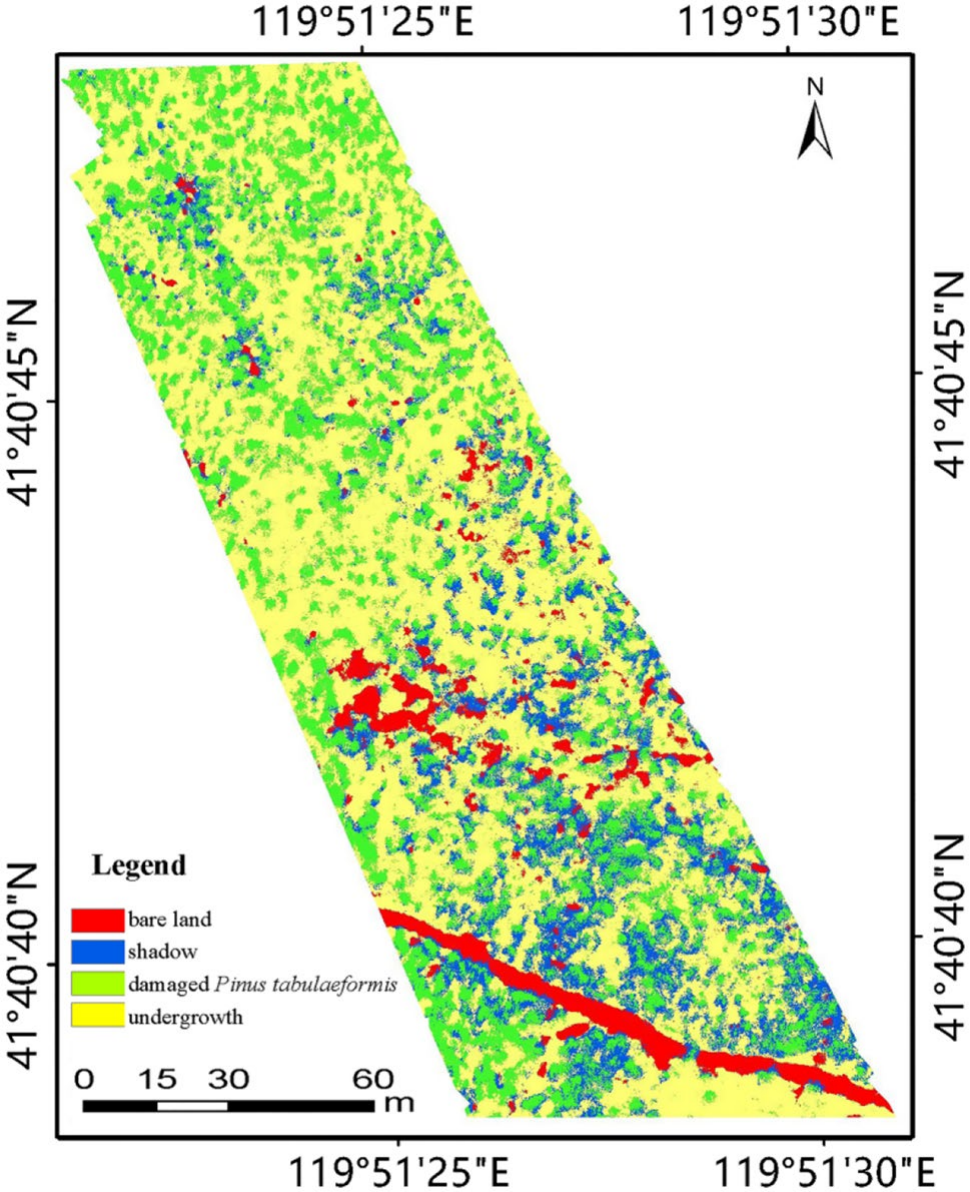
SVM classification map with the G-RBF kernel (20, 0.5)

Table 1 summarizes the a part of classification results of the G-RBF under different parameter setting of grid search. Numerous experiments showed that, as C decreases, the OA first is invariable and then decreases, and finally tends to be the same. And as $\gamma$ decreases, both OA and evaluating indicator has a certain degree of decrease. Furthermore, under the same classification accuracy, the program running time is increase with the increase of $\gamma$. Therefore, considering both the classification accuracy and program running time (about 20 min), the SVM classification of the UAV-based hyperspectral data in the entire study area was performed using the parameter combination (20, 0.5) in the G-RBF kernel function (Fig. 1).

**Table 1:** A part of classification results of G-RBF under different parameters

Kernel Function	Parameters		OA/%	Kappa	CADP/%
	C	$\gamma$			
G-RBF	100	0.5	89.3258	0.8514	76.87
	80	0.5	89.3258	0.8514	76.87
	60	0.5	89.6067	0.8554	77.44
	40	0.5	89.6067	0.8554	77.44
	*20*	*0.5*	*90.4494*	*0.8673*	*79.23*
	20	1	89.6067	0.8554	77.44
	15	0.5	90.4494	0.8673	79.23
	15	1	90.4494	0.8673	79.23
	10	0.5	90.4494	0.8673	79.23
	5	0.5	90.4494	0.8673	79.23

*The italic fonts represent the best classification result

### Optimization results of two EPFs

We compared and analyzed the probability optimization effects of two types of EPFs with two types of guidance images under different parameter settings. For the JBF, we set the filter kernel size (${\sigma }_{d}$) and the proportion of the weight change in the local window (${\sigma }_{r}$). Based on previous studies, ${\sigma }_{d}$ was set to 1, 2, 3, and 4, and ${\sigma }_{r}$ was set to 0.01, 0.1, 0.2, and 0.4, respectively. For the GF, we set the window radius ($r$) and control gradient changes ($\epsilon$). Specifically, $r$ was set to 1, 2, 4, and 8, and $\epsilon$ was set to 0.01^2^, 0.1^2^, 0.2^2^, and 0.4^2^, respectively. Due to the category of the damaged *Pinus tabulaeformis* we focused on, the optimal parameters of the EPFs were determined with the accuracy of the damaged *Pinus tabulaeformis* extraction.

#### Evaluation of JBF effects

Table 2 summarizes the MSSIM evaluation results of the initial probability maps corresponding to the damaged *Pinus tabulaeformis* category after applying JBF. It can be seen that, with the RGB image as the guidance image, the MSSIM is higher than that of the PCA false-color image as the guidance image under the same parameter settings. Regardless of which guidance image was selected, when ${\sigma }_{d}$ is fixed, as ${\sigma }_{r}$ increases, the MSSIM gradually decreases; similarly, when ${\sigma }_{r}$ is fixed, as ${\sigma }_{d}$ increases, the MSSIM gradually decreases.

**Table 2:** MSSIM results of JBF under different parameters

Guidance image	MSSIM	${\sigma }_{r}=0.01$	${\sigma }_{r}=0.1$	${\sigma }_{r}=0.2$	${\sigma }_{r}=0.4$
RGB image	${\sigma }_{d}=1$	*0.8754*	0.8654	0.8451	0.8348
	${\sigma }_{d}=2$	0.8593	0.8537	0.8389	0.8386
	${\sigma }_{d}=3$	0.8122	0.8117	0.8014	0.7909
	${\sigma }_{d}=4$	0.7763	0.7690	0.7554	0.7483
PCA false color image	${\sigma }_{d}=1$	*0.8478*	0.8425	0.8375	0.8269
	${\sigma }_{d}=2$	0.8217	0.8168	0.8102	0.8072
	${\sigma }_{d}=3$	0.7986	0.7945	0.7864	0.7735
	${\sigma }_{d}=4$	0.7513	0.7479	0.7412	0.7358

*The italic fonts indicate optimized precision

**Fig. 2:**
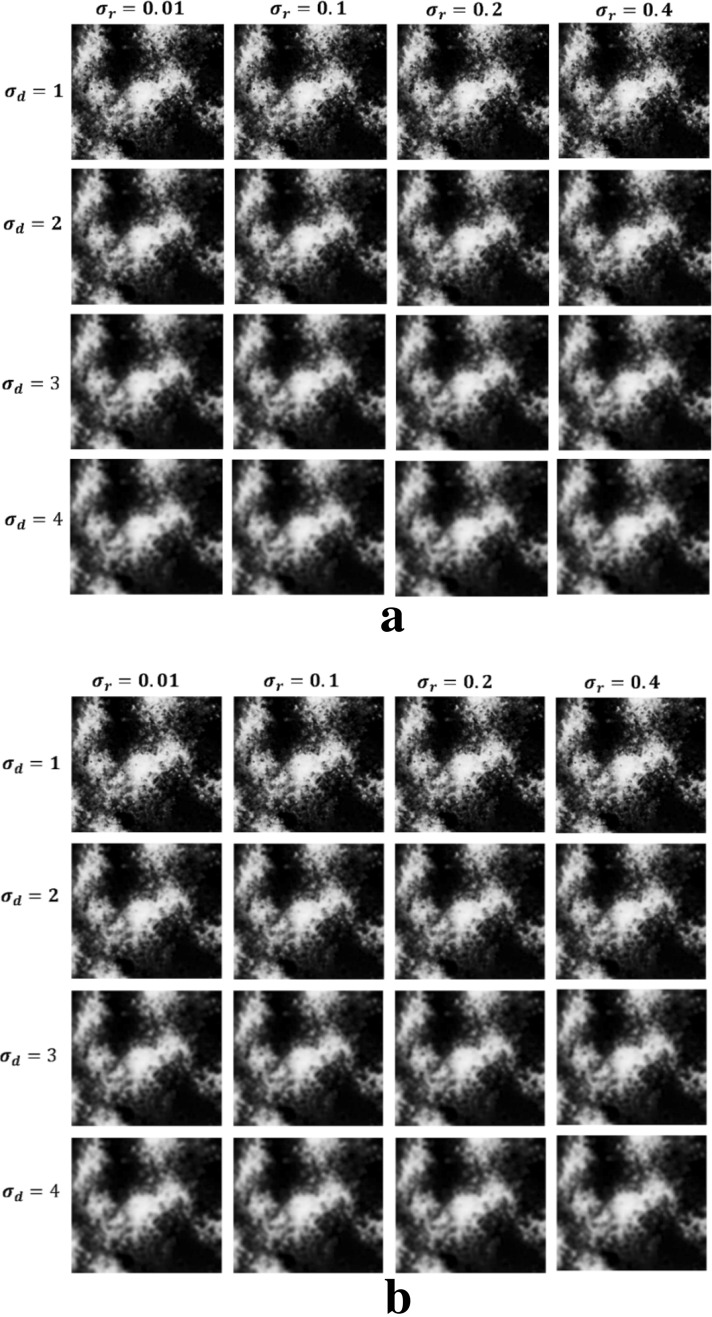
Optimized probability map obtained by JBF (the type of damaged *Pinus tabuliformis*). **a** The guidance image is RGB image, **b** the guidance image is PCA false-color image. *JBF* the joint bilateral filter

Figure 2 shows the effect of the JBF on the initial probability map of the damaged *Pinus tabulaeformis*. As can be seen, when ${\sigma }_{d}$ and ${\sigma }_{r}$ are small, the image sharpness is higher, and the edge information is clearer; that is, the edge-preserving effect is stronger. Conversely, when ${\sigma }_{d}$ and ${\sigma }_{r}$ are large, the image sharpness is lower, and the edges are blurred; that is, the edge-preserving effect is weaker.

The smaller the values of ${\sigma }_{d}$ and ${\sigma }_{r}$, the larger the corresponding MSSIM, and the more effectively the EPF maintains the guidance image. When ${\sigma }_{d}$ = 1, the edge-preserving effect of the image is better than that with other values of ${\sigma }_{d}$; specifically, when ${\sigma }_{d}$ = 1, the MSSIM values corresponding to ${\sigma }_{r}$ = 0.01 and ${\sigma }_{r}$ = 0.1 are both large and have a slight difference, and considering the operating rate at the same time, ${\sigma }_{d}$ = 1 and ${\sigma }_{r}$ = 0.1 was determined as the optimal parameter combination for the JBF.

#### Evaluation of GF effects

Table 3 summarizes the MSSIM evaluation results of the initial probability maps corresponding to the damaged *Pinus tabulaeformis* category after applying the GF. In the case of various parameter settings with the RGB image as the guidance image, the MSSIM yielded results superior to those obtained using the PCA false- color image as the guidance image. Moreover, regardless of the guidance image selected, when $r$ is constant, the MSSIM appears to increase and then decrease with changes in $\epsilon$. In most cases, the MSSIM has the largest value when $\epsilon ={0.1}^{2}$. When $\epsilon$ is constant, the MSSIM shows a significant downward trend as $r$ increases.

**Table 3:** MSSIM results of GF under different parameters

Guidance image	MSSIM	$\epsilon ={0.01}^{2}$	$\epsilon ={0.1}^{2}$	$\epsilon ={0.2}^{2}$	$\epsilon ={0.4}^{2}$
RGB image	$r=1$	0.9187	***0.9227***	0.9064	0.9015
	$r=2$	0.8594	0.8646	0.8513	0.8474
	$r=4$	0.7482	0.7239	0.7144	0.7011
	$r=8$	0.6289	0.5955	0.5626	0.5449
PCA false-color image	$r=1$	0.9097	*0.9118*	0.9005	0.8956
	$r=2$	0.8437	0.8513	0.8447	0.8331
	$r=4$	0.7048	0.7165	0.6946	0.6632
	$r=8$	0.6090	0.6132	0.5518	0.5053

*The italic fonts indicate optimized precision

**Fig. 3:**
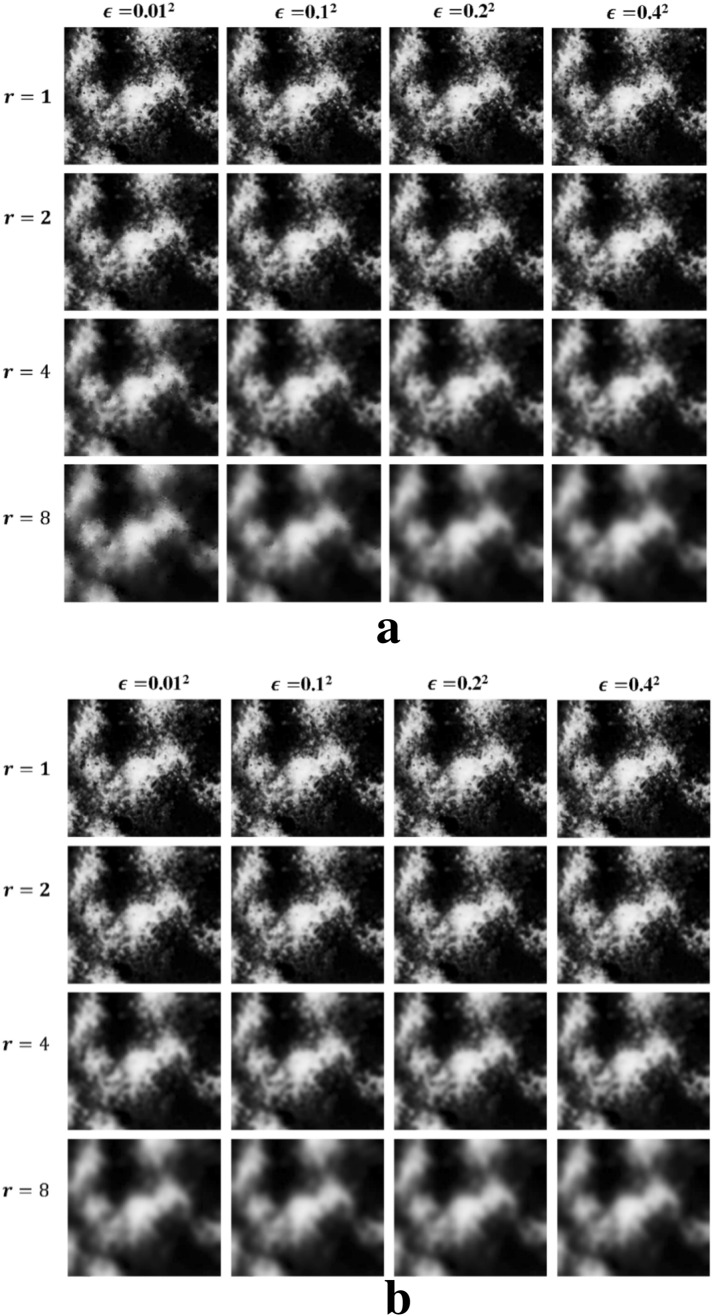
Optimized probability map obtained by GF (type of damaged *Pinus tabuliformis*)*. a* The guidance image is RGB image, **b** the guidance image is PCA false-color image. *GF* the guided filter

As shown in Fig. 3, when $r$ is large, the image sharpness is reduced, and the discrepancy increases gradually with $\epsilon$. On the contrary, when $r$ is small, the image sharpness does not change significantly with $\epsilon$. Therefore, the larger the value of $r$ that controls the size of the local window, the larger the range in which the filtering algorithm performs averaging and the smoother the result. Further, $\epsilon$ controls the gradient preserving effect; the larger the value of $\epsilon$, the larger the smoothing factor of the overlay, and the effect is minimal when the window is small.

According to Tables 2 and 3, the MSSIM for the GF under different parameter settings remains the same as that for the JBF. The smaller $r$, the larger the corresponding MSSIM. However, the optimal result is obtained when $\epsilon ={0.1}^{2}$, and the EPF preserves the guidance image better. When $r=1$, the MSSIM is high and the fluctuation is not large. Note that as $\epsilon$ is in the denominator in Eq. 9, it ultimately affects the image gradient preserving ability; the smaller $\epsilon$, the stronger the edge preserving ability. However, the algorithm is relatively smooth in this case. After determining $r$ = 1, we compared $\epsilon ={0.01}^{2}$ and $\epsilon ={0.1}^{2}$; $\epsilon ={0.1}^{2}$ was found to correspond to a better edge-preserving ability.

#### Comparison of the two filtering methods

By comparing and analyzing the filtering effects of the two filters and their MSSIM changes, we found that their sharpening effects were better when the control filter kernel (${\sigma }_{d}$ and $r$) and ambiguity (${\sigma }_{r}$ and $\epsilon$) parameters were higher; however, the edge-preserving ability decreased. This decrease occurred because a large filter kernel, or ambiguity, may cause excessive smoothness, and some small-scale targets may be misclassified. With increasing ${\sigma }_{d}$ and ${\sigma }_{r}$ in the JBF, and increasing $r$ and $\epsilon$ in the GF, the increase in the kernel size (${\sigma }_{d}$ and $r$) reduced the edge-preserving effect much more than the effect of ambiguity (${\sigma }_{r}$ and $\epsilon$) on it. When the filter kernel (${\sigma }_{d}$ and $r$) was fixed and the ambiguity (${\sigma }_{r}$ and $\epsilon$) was adjusted, MSSIM was almost unchanged, and the optimized probability maps did not change significantly; when the filter kernel (${\sigma }_{d}$ and $r$) was adjusted, the change in MSSIM was relatively large, the optimal probability maps could also show edge changes. Figures 2 and 3 show the substantial changes of partial edge enhancement of the two types of EPFs with different parameter settings in the case of the RGB image as the guidance image.

For the selection of the guidance image, through the above-mentioned analysis, for both JBF and GF, the MSSIM is higher when the RGB image is used as the guidance image compared with when the PCA false-color image is used as the guidance image. As the guidance image, the RGB image has good texture and spatial characteristics compared with the PCA false-color image, and its intra-class difference is reduced more effectively. The edge information of the feature is highlighted, which is consistent with the evaluation effect of the MSSIM. EPF optimization with the RGB image as the guidance image yields better results, especially in the case of GF. Based on the results shown in Tables 3 and 4, the optimal parameters for the JBF were set to ${\sigma }_{d}=1$, ${\sigma }_{r}=0.1$, and the optimal parameters for the GF were set to $r=1$, $\epsilon ={0.1}^{2}$.

**Table 4:** Comparison of classification results obtained by original SVM and optimized SVM

	Filter	OA /%	Kappa	CADP/%
SVM	/	90.45	0.8673	79.23
Optimized SVM	Joint Bilateral Filter	91.63	0.8921	86.73
	Guided Filter	93.17	0.9053	88.97

### Extraction of tree crowns of damaged *Pinus tabulaeformis*

**Fig. 4:**
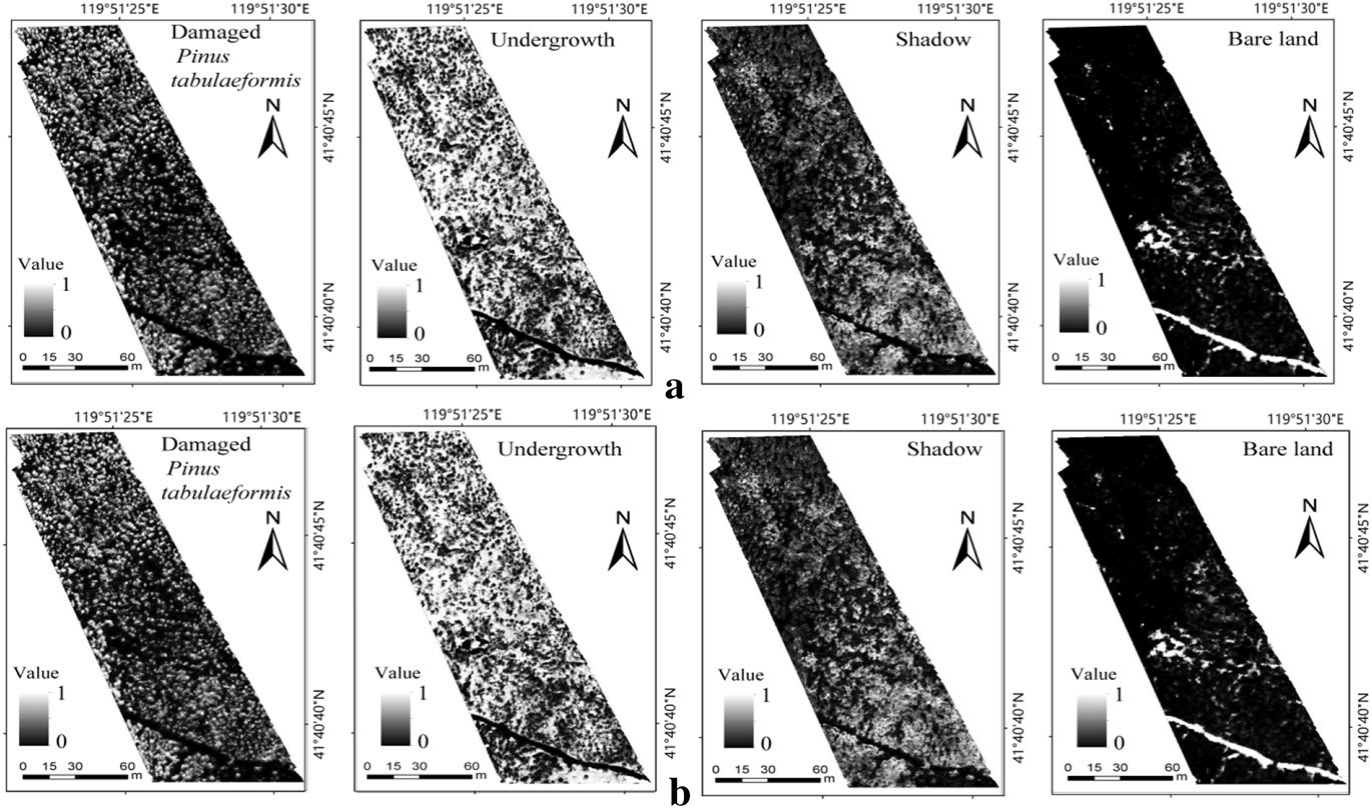
Optimized probability maps for the four classification types under different EPFs with **a** JBF and **b** GF. *JBF* the joint bilateral filter, *GF* the guided filter

By analyzing and comparing the SVM classification method and EPF algorithms, the best spectral-spatial classification framework for tree crowns extraction of damaged *Pinus tabulaeformis* was completed using the G-RBF (20, 0.5) kernel function with the RGB image as the guidance image. Furthermore, the optimal parameter combinations for the initial probability maps of the SVM classification were determined to be ${\upsigma }_{\mathrm{d}}$ = 1, ${\upsigma }_{\mathrm{r}}$= 0.1 for the JBF and $\mathrm{r}$= 1, $\upepsilon ={0.1}^{2}$ for the GF. After determining the category of the maximum probability value of each pixel, the classification of all the pixels was completed, and the classification results of the entire area were evaluated using 1250 verification samples from the original SVM classification. Figure 4 shows the optimized results for the four classification types with different EPFs.

**Fig. 5:**
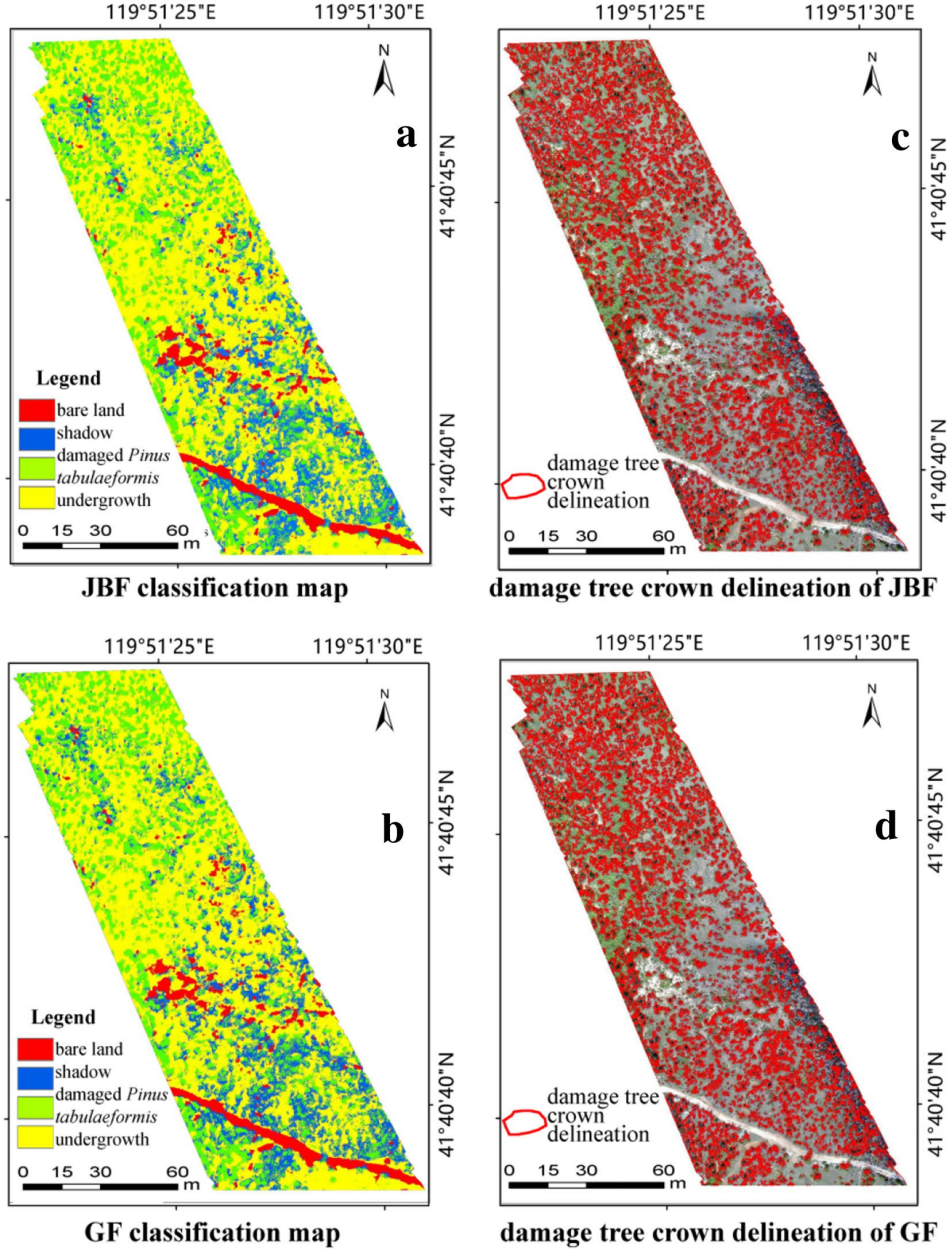
Final classification results of spectral-spatial classification based on optimized SVM. *JBF* the joint bilateral filter, *GF* the guided filter

As shown in Table 4, the classification results of the optimized SVM proposed in this study show higher OA and Kappa compared to the original SVM classification results. Comparing the optimized SVM classification results based on the two EPFs, SVM based on GF has higher classification accuracy and the OA reaches 93.17%, which is consistent with the evaluation results of MSSIM of the guidance image obtained using the two filter methods. Thus, GF is favorable for spatial information retention of the RGB image, and the edge-preserving effects of various features are better. Correspondingly, after optimization of the SVM classification results using GF, the OA is higher, which means that the edge-preserving strength will directly affect the final classification results. Figure 5 shows the classification results obtained using the proposed spectral-spatial classification.

In the above-mentioned final classification results, the identification of damaged *Pinus tabulaeformis* was completed after extracting the range corresponding to the damaged *Pinus tabulaeformis* category. The classification accuracy of damaged *Pinus tabulaeformis* extraction using the SVM spectral-spatial classification method based on the EPF (86.73% for JBF and 88.97% for GF) was higher than that using the original SVM classification (79.23%), and the optimization effect was obvious. The effect of GF was favorable, which corresponded to the strong edge-preserving ability of the RGB image. Compared with the overall optimized effect, the edge of a single *Pinus tabulaeformis* is more precise and the edge-preserving ability has a stronger influence on the classification results of *Pinus tabulaeformis*. Therefore, the optimization intensity of classification accuracy of *Pinus tabulaeformis* is higher.

## Discussion

### Using UAV-based hyperspectral images for damaged tree crown extraction

For forest surveys, precise tree crown extraction is the first step to achieve research on forest structure parameters. It can provide fundamental support for forest healthy diagnosis and damage assessment. *D.tabulaeformis* is a type of defoliation pest. When extensive damage occurs, the defoliation rate of the entire plant can reach 100%. In this regard, damaged trees show complex shapes and crown contours. Hence, this phenomenon provides harsher conditions for precise tree crown extraction.

UAV-based hyperspectral imaging technology can provide both spatial geometric information, such as the tree crown shape, size, and texture; the relationship between adjacent tree crowns; and exact spectral information to accurately detect small changes in the pigmentation, water content, and structure of the tree crown. Forestry has benefitted from the use of this technology [29] for tree species classification [30], health monitoring [31], and biodiversity assessment [32]. However, UAV-based hyperspectral images have not been widely applied to forest-health detection compared with other data sources. Current studies focus on first detecting tree crowns and then assessing tree health through crown damage symptoms or crown-level spectral information [33–35]. This process increases the amount of data processed by the algorithm runs and may generate double-errors.

By combining the advantages of UAV-based hyperspectral image data, we tried to extract the range of damaged tree crowns directly using hyperspectral and RGB images acquired by a UAV-based imaging system. First, owing to the high spectral resolution, we could accurately distinguish objects with similar color or texture features, such as severely damaged tree crowns and soil, healthy leaves, and undergrowth. Due to high sensitivity to damaged trees, especially to slight and moderate damage, hyperspectral images provide tree crown extraction results with higher accuracy compared to high-spatial-resolution remote sensing images. Compared with the process of first extracting the tree crown and then identifying the disease, the use of UAV-based hyperspectral images effectively overcomes the mismatch between the spectral data (always acquired by field spectral devices, the commonly used are FieldSpec® which are produced by Analytical Spectral Devices., Inc.) and the high-resolution images. Second, RGB images used as guidance maps provide edge structure information to improve the extraction accuracy. This method takes advantage of the spectral information of hyperspectral data and the spatial characteristics of RGB data. The UAV is sufficiently flexible to meet the needs of practical applications. Therefore, the proposed method can be used to extract the damaged forest range automatically and accurately, which is beneficial for rapid and effective assessment of forest damage in large areas.

### Spectral–spatial classification framework based on SVM and EPF for damaged tree crown extraction

Considering both high spatial and spectral resolution of UAV-based hyperspectral, in order to improve the classification performance of the hyperspectral images further, many studies have focused on the spectral–spatial classification framework. Whether in the direction of feature extraction algorithm construction or spectral classification result optimization, hyperspectral image spectral–spatial classification is widely used for many applications [36, 37].

In the first step of the proposed method, the SVM pixel-wise spectral classifier is applied to the hyperspectral image, and the classification results are represented as the initial probability map. The SVM algorithm can distinguish the land cover types, and the hyperspectral image can capture detailed spectral information. However, tree shadows, branches, and underlying objects may lead to the mixed pixel problem, especially for the edges of damaged conifers. Furthermore, the illuminated side and shaded side of each tree crown may produce different spectral signatures, leading to the double-side illumination problem. Thus, although the pure SVM classifier fuses the spectral and spatial features by kernel combination to some extent, it cannot satisfy the accuracy requirements of damaged conifer tree crown extraction and delineation [38, 39]. Hence, EPFs were used to extract the spatial features after using the SVM to construct the spectral–spatial classification framework. The EPF algorithms aim to achieve local optimization of the initial probability map compared with other methods and thus are extremely suitable for damaged tree crown edge delineation. Both the JBF and GF have the function of a joint EPF, which combines the edge structure information of the guidance image while filtering one image. Some errors exist in the edge obtained in the first step, and these errors can be reduced by processing the RGB image with the EPF algorithms.

Comparison of the results of spectral–spatial classification and pure SVM classification showed that the spectral–spatial classification method improved the extraction accuracy of damaged tree crowns (the CADP of GF reached 88.97%, which is 9.74% higher than that of pure SVM; see Table 4). Thus, the proposed method is computationally efficient and will be useful in practical applications.

### Factors Influencing damaged tree crown extraction

Forestry remote sensing detection results always involve some degree of uncertainty because of the complexity of the forest structure, and the same is true for forest health monitoring. As far as the objective factors are concerned, the complexity of the forest structure, the condition of the terrain, natural conditions such as light and wind speed, and the flight altitude of the UAV have certain effects on the classification and tree crown extraction results. From the aspect of efficient algorithms, the proposed spectral–spatial classification framework requires the selection of parameters and guidance maps, and the resolution differences between guidance maps and classification maps, all of which will affect the final classification results. In addition to the classification accuracy, the computational efficiency is an important consideration.

The classification accuracy was clearly improved to a certain extent by using the proposed spectral–spatial classification framework, especially the CADP, as shown in Table 4. Although the above-mentioned influencing factors are unavoidable, the process of selecting algorithm parameters involves adaptation to the objective environmental impact factors. Thus, according to every parameter of the algorithm, the proposed spectral-spatial classification framework can be adapted to different environments to a certain extent and can be considered as a universal framework for different forests and conditions.

### Further application

As can be seen from Fig. 5, damaged tree crowns can be mapped after using the proposed spatial-spectral classification framework extracted the damaged trees. The mapped profiles represent the important regions of interest (ROIs) for forest disease detection, identification, and damage-level analysis. At the same time, the ROIs are an indispensable part of remote sensing, especially hyperspectral remote sensing analysis.

**Fig. 6:**
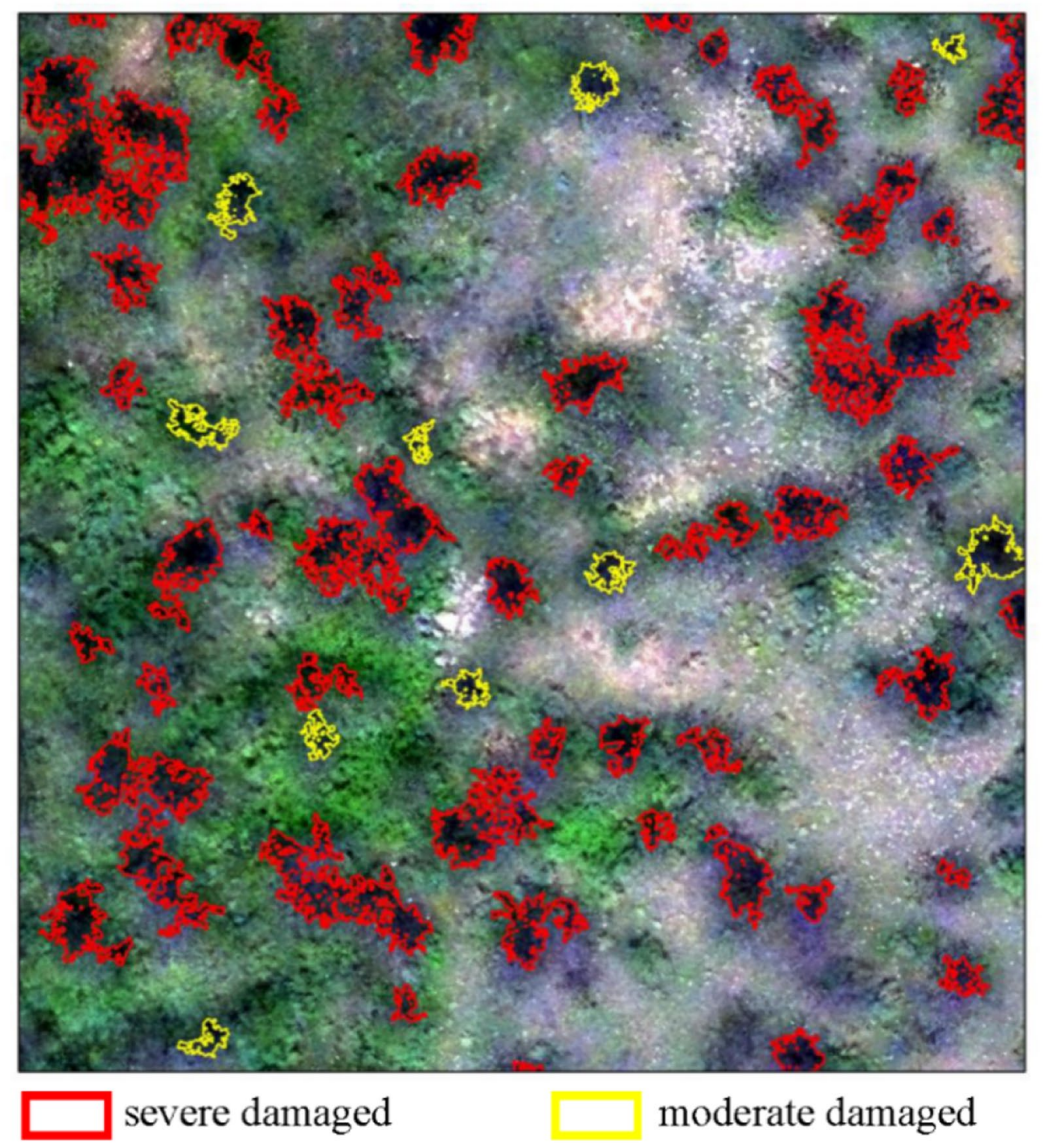
The assessment of *D. tabulaeformis* damaged level by using the proposed spatial-spectral classification framework to extract damaged tree crown automatically

In “Using UAV-based Hyperspectral Images for Damaged Tree Crown Extraction Section”, we discussed that the proposed spectral-spatial classification framework can be directly used to classify and extract the damaged tree with different damage levels. Furthermore, with the spectral disease indices (SDIs) developments, the proposed framework also can be used as the ROIs construction methods first, and then, the SDIs can be selected to determine the damage types or severity. Combined with the research the research presented by Zhang et al. [40], the damaged tree crown extraction can be applied before extracting average reflectance of individual *D.tabulaeformis* damaged trees. This step can replace the manual drawing of tree crown ROIs, and realize the automatic assessment of defoliation. Because this study and the previous research use the same set of data, we used the piecewise model in Zhang’s research to realize defoliation level assessment. Figure 6 shows the damage level assessment results. This is a typical further application of damaged tree crown extraction. Basic automatic analysis can be used for not only forest-damage analysis, but for all types of forest structural parameters, and even for carbon sinks, net primary productivity, and biomass estimation, as long as crown automatic extraction is realized.

### Deficiencies and outlook

Traditional manual surveying of forest health is time-consuming, laborious, and prone to errors and omissions. Low-altitude remote sensing and hyperspectral detection can identify subtle changes in tree crowns through detailed spectral changes. The spectral–spatial classification framework and UAV-based hyperspectral images used in this study yielded good results for damaged tree crown extraction. However, this study has the following limitations. First, the research area selected for this study was an artificial pure *Pinus tabulaeformis* forest, which is only damaged by *D. tabulaeformis*. However, in most cases, forests suffer from many types of pests and diseases. Effective distinction between damage by *D. tabulaeformis* and that due to other hazards requires further analysis. Second, we did not identify the tree crowns of healthy *Pinus tabulaeformis* in this study because there were almost no healthy *Pinus tabulaeformis* in the study area, which may raise some concerns. Finally, not all the crowns of damaged *Pinus tabulaeformis* that we extracted were individual tree crowns. In the case of a single tree, the crown that we extracted was an individual tree crown. In the case of multiple aggregated trees, the crown that we extracted was the outermost edge of the crown of the multiple trees.

Considering the application requirements and the limitations discussed above, further improvement may be a prerequisite for future analysis. On the one hand, the method can be validated by obtaining study areas containing healthy and damaged *Pinus tabulaeformis* as conditions permit. On the other hand, by using time-series image data, which can satisfy the biological and ecological characteristics of pests and diseases or the phenological characteristics of vegetation, different tree species as well as different diseases and insect pests can be distinguished. Furthermore, for the damaged *Pinus tabulaeformis*, the spectral characteristics of different degrees of damage or defoliation obtained from hyperspectral images and high-spatial-resolution images can be used to assess the degree of damage and extract the individual tree crowns.

## Conclusion

In this study, UAV-based hyperspectral images were used to extract tree crowns damaged by *D. tabulaeformis* based on a spectral–spatial classification framework. This classification framework achieved the objective of precise damaged tree crown extraction by optimizing the SVM pixel-wise classification results using an EPF. The final tree crown extraction accuracy reached 86.73% and 88.97% under the JBF and GF algorithms, respectively. The results showed that (1) UAV-based hyperspectral images can be used as the sole data source to extract damaged tree crowns; (2) the damaged canopy can be feasibly identified by selecting appropriate parameters of the SVM (for G-RBF, the parameter combination of (20, 0.5) provides higher classification accuracy and applicability, with OA = 90.45%, Kappa = 0.8673, and CADP = 79.23%); (3) the optimized SVM based on the proposed EPF can effectively improve the CADP (the extraction accuracy of damaged tree crowns is 7.50%–9.74% higher than that in the case of the traditional SVM classifier); (4) by using the RGB image as the guidance image, better EPF optimization can be achieved and the CADP can be improved with more detailed spatial information. To the best of our knowledge, this study is one of only a few in which a UAV-based hyperspectral image has been used to extract tree crowns damaged by *D. tabulaeformis*. Moreover, the proposed classification method can effectively extract damaged tree crowns; hence, it can serve as a reference for future studies on both forest health monitoring and larger-scale forest pest and disease assessment.

## Materials and methods

### Study area

**Fig. 7:**
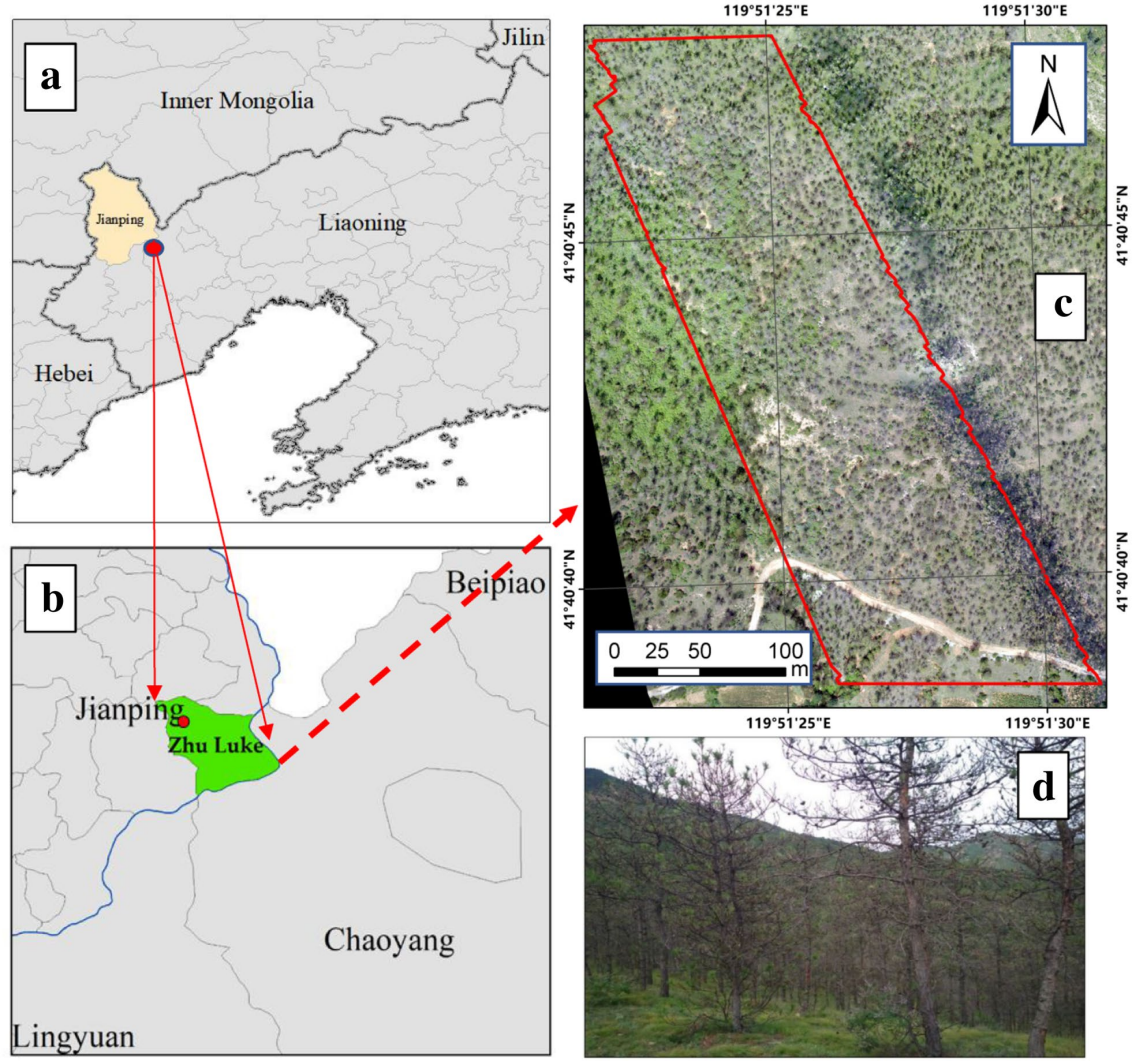
The location of the study area. **a**, **b** Location of the study area in Liaoning Province. **c** Distribution of the study area on RGB image acquired from UAV on August 5, 2016. **d** Severe damaged trees’ photo in the field. The defoliation rate of this plot reached 100%, and almost 90% of plots are the same in the study area

The study area is located in Zhu Luke, Jianping County of Liaoning Province, China (41°19′–41°23′N, 119°14′–120°03′E, Fig. 7). The area has a temperate continental climate with dry, windy spring and autumn seasons and a rainy summer. The annual average temperature is 7.6 °C, the annual average precipitation is 614.7 mm. Vegetation mainly consists of forest and shrubland. The forest area in Jianping County is 21.83 × 104 ha, and pure pine species occur in 50% of the total forest area.

According to the statistics of the local forest protection station, the average annual area damaged by *D. tabulaeformis* in the study area is 176,000 ha. According to the forest investigate records, which provided by the Station of Forest Protection of Jianping County, there are few other biotic or abiotic stress factors in the study area. And the simultaneous trapping experiment of *D.tabulaeformis* showed that the larva population density has reached severe damaged level. Based on the above situations and the data from 2014 shows that defoliation of *Pinus tabulaeformis* in the study area was mainly caused by *D.tabulaeformis* [40].

### Field data acquisition and preprocessing

The field survey was conducted in August 2016 in the northwest region Zhu Luke town. Specifically, an area of 29,789 m^2^ was established for continuous monitoring of the pure *Pinus tabulaeformis* forest, with trees suffering from different damage degrees of *D.tabulaeforms*. The average altitude of the study area is about 495 m and is relatively flat with no valleys or ravines. The coordinates of the corner points of this area were recorded by a handheld differential global positioning system (DGPS, Version S760, South Surveying & Mapping Technology Co., Ltd. Guangzhou, China) with sub-meter accuracy. The diameter at breast height (DBH) and the heights of each tree for which DBH ≥ 10 cm were measured, and the location information of all damaged trees was recorded simultaneously. All the records of DGPS were exported in a.txt file, then converted to Excel to build a.shp file.

For each sampled tree, the defoliation rate were calculated by survey the standard branches (which can can roughly represent the average defoliation of the tree) as the damaged level divided criterion (The detailed criterion were published in Zhu et al. [41]). The standard branches, which clipped from the upper, middle and lower three layers from four directions, tree were clipped and counted the total number of pine needles and the damaged needles. As Du [42] published, the degree of loss level were set as 0, 25, 50, 75 and 100%. Final defoliation rate were calculated by Eq. (1):

1 $$\left\{ {\begin{array}{*{20}c} {p_{i} = \frac{{0\% *N_{0\% } + 25\% *N_{25\% } + 50\% *N_{50\% } + 75\% *N_{75\% } + 100\% *N_{{100{\text{\% }}}} }}{{N_{0\% } + N_{25\% } + N_{50\% } + N_{75\% } + N_{{100{\text{\% }}}} }}} \\ {P = \frac{{\sum {P_{i} } }}{3}} \\ \end{array} } \right.$$

where $\mathrm{P}$ is the defoliation rate of an individual sampled tree, ${\mathrm{P}}_{\mathrm{i}}$ denotes the defoliation rate of a standard branch of every layer (upper, middle, and lower) of a tree, and ${\mathrm{N}}_{\mathrm{n\%}}$ represents the number of needles as the leaf loss of n%.

### Image data acquisition and preprocessing

A UAV-based hyperspectral imaging system was used for image data acquisition. The overall UAV-based system employed in this study consisted of a DJI Spreading Wings S1000 + multi-rotor octocopter-UAV (DJI, Shenzhen, China), a UHD 185 snapshot imaging spectrometer in the visible-NIR wavelength range (Cubert GmbH, Ulm, Baden-Württemberg, Germany), and a Sony DSC-QX 100 digital camera (equipped with a 13.2 mm × 8.8 mm Exmor R sensor with a resolution of 20.2 megapixels). The UHD 185 snapshot imaging spectrometer can simultaneously capture the low spatial resolution hyperspectral cube and high-spatial resolution panchromatic image in one spectral channel. The main parameters of the UHD 185 are listed in Table 5.

**Table 5:** Main parameters of the UHD 185 imaging spectrometer (provided by the manufacturer)

Parameters	Value	Parameters	Value
Total weight	470 g	Wavelength range	450–950 nm
Digitization	12 bits	Sampling interval	4 nm
Field of view	19°	Spectral resolution	8 nm at 532 nm
Cube resolution	1.0 megapixels	Spectral channels	125

UAV-based data acquisition was conducted in the test areas of Zhu Luke on August 5, 2016, under sunny and windless conditions. Given terrain, vegetation condition, and coverage of the study area, the flying height was set to 100 m. The focal length of the digital camera (equivalent to 35 mm focal length) and the hyperspectral camera were set as 28 mm and 23 mm separately, and the optimal resolution of digital camera was 5472 × 3648. Totally, four flights were performed to cover the whole area. And each flight, the image forward and side overlaps were set to 70 and 60%, respectively. The hyperspectral and RGB images were acquired simultaneously. The RGB images, which were used to indicate the digital images acquired using a Sony DSC-QX100 digital camera, had three bands: red, green, and blue.

A total of 14,082 images covering the study area were acquired from the UHD 185, and 7726 images acquired during takeoff, landing, and turning were deleted from each flight. In total, 6356 effective images covering the test area were obtained. These include panchromatic images (JPG format, 1000 × 1000 pixels) and hyperspectral images (cub format, 50 × 50 pixels), where panchromatic and hyperspectral images correspond to each other in a uniform geographic range. The estimated ground sampling distances were 0.028 and 0.56 m for panchromatic and hyperspectral images, respectively. The preprocessing step mainly includes image fusion, mosaic, geometric correction, and radiometric calibration. The image fusion and format conversion of original panchromatic images and hyperspectral images were performed by using the image processing software Cube-Pilot in UHD185 hyperspectral imager. The format of the hyperspectral cubes was converted from.cub to.tif and the size was changed from 50 × 50 pixels to 1000 × 1000 pixels. The resolution of hyperspectral images was 0.028 m through image fusion. The panchromatic images were used to align photos, build a dense cloud, and build a mesh with interpolation POS information in the Agisoft PhotoScan Professional Pro (Version 1.1.6, Agisoft LLC, Russia). On this basis, we used the hyperspectral cubes as substitutes for the panchromatic images in PhotoScan, and built the texture using the pixel values from the hyperspectral cubes instead of the pixel values of the panchromatic images. Through above steps, geometric correction completed and the hyperspectral orthophoto images were generated. Radiometric calibration was directly performed for hyperspectral orthophoto images after geometric correction. The main principle and equations, which are about the radiometric calibration, have already been described in detail in [43]. A total of 2087 effective RGB images and original POS data were used to generate the RGB orthophoto images. This process was completed in PhotoScan, through aligning photos, building dense cloud, building mesh and building textures steps. The RGB images were resampled to 0.028 m after mosaic, the same as hyperspectral images. The specific preprocessing methods of the hyperspectral images have already been described in detail by the co-author in a previous article [40].

### Spectral-Spatial classification framework construction

**Fig. 8:**
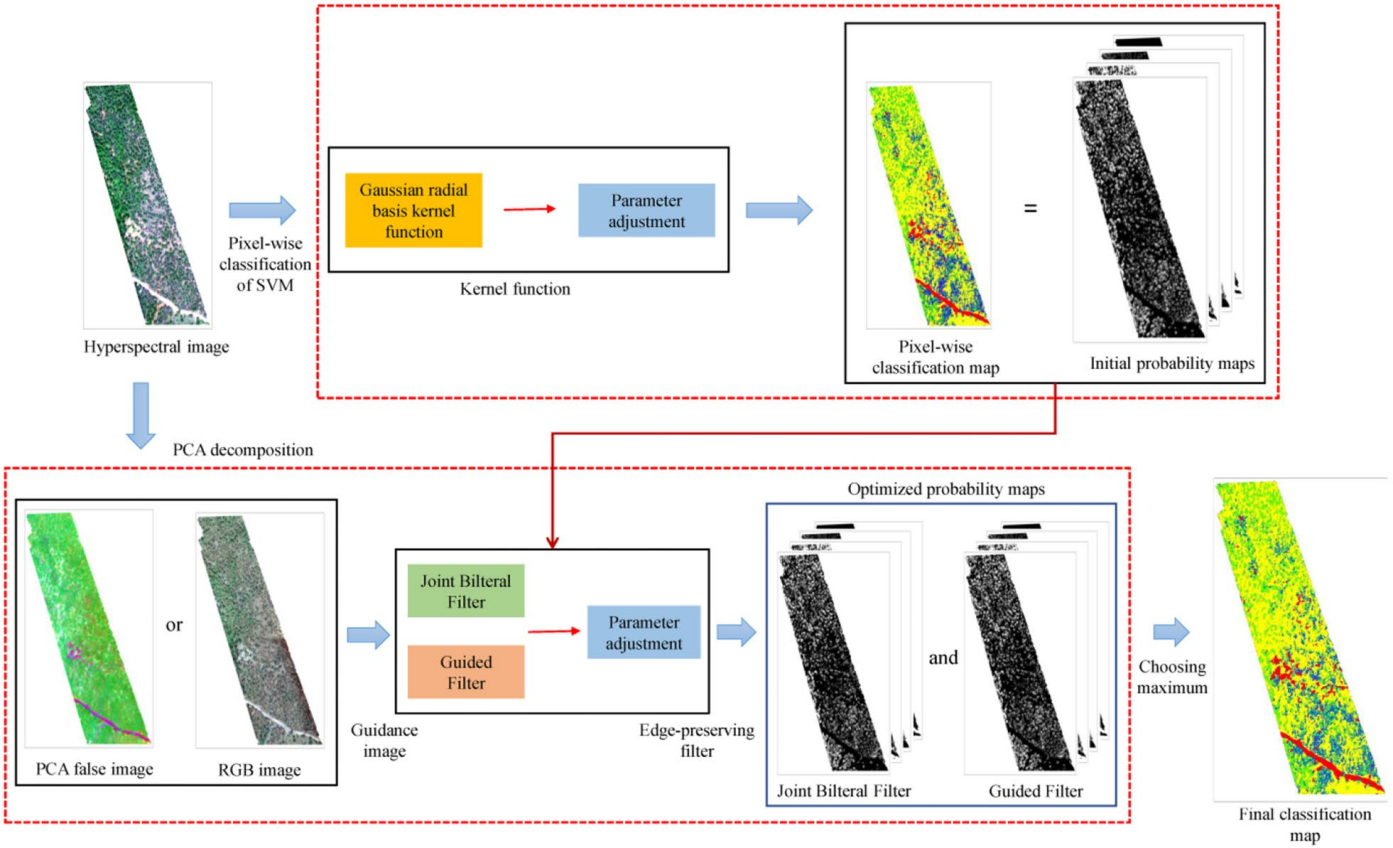
Schematic of the proposed spectral-spatial classification framework. PCA false-color image: principal component analysis false color image

Figure 8 shows the steps involved in the construction of the spectral-spatial classification framework for UAV-based hyperspectral images. This process roughly involves (i) construction of initial probability maps for discriminating classes by the SVM pixel-wise spectral classifier using the original bands of the hyperspectral images, (ii) optimization of the spectral classification results by the EPF algorithms, and (iii) mapping of the final classification results.

#### Initial probability map construction by SVM classifier

The SVM classifier is a supervised, non-parametric, statistical learning technique [44] that aims to find an optimal hyperplane for solving the class separation problem [45]. SVM is a widely used pixel-wise classifier for hyperspectral image classification. The basic idea of SVM classifier is to improve the dimension and linearize the data. In essence, it nonlinearly transforms the defined inner product function, converts the entire image into high-dimensional space, performs linear fitting, and then determines the optimal linear classification surface. The inner product function is the kernel function, which directly affects the final classification results [8].

Commonly used kernel functions include the linear kernel function, polynomial kernel function, Gaussian radial basis kernel function, and sigmoid kernel function. Compared with other kernel functions and other application researches, the Gaussian radial basis kernel function (G-RBF) has certain advantages in classification and recognition. The G-RBF can effectively deal with the relationship of sample nonlinearity and map it to higher dimensional space. It has less numerical complexity and is easy to adjust, especially for high-dimensional classification features. Therefore, the G-RBF is selected for SVM classification in this study, the detailed kernel function show in Eq. (2).

2 $$\left\{ {\begin{array}{*{20}c} {f(x) = {\text{sgn}} [\sum\nolimits_{i = 1}^{k} {\alpha_{i}^{*} y_{i} K(x_{i} ,x) + b^{*} } ]} \\ {K(x_{i} ,x) = \exp [ - \frac{{\left\| {x - x_{i} } \right\|^{2} }}{{\gamma^{2} }}],\gamma > 0} \\ \end{array} } \right.$$

The constraint condition in here is $\sum\nolimits_{i = 1}^{k} {\alpha_{i} y_{i} = 0,} 0 \le \alpha_{i} \le C,i = 1,2,...k$, C is a constant that represents the penalty coefficient of SVM; $k(x_{i} ,x)$ is the G-RBF kernel function.

When using SVM with the G-RBF kernel, two parameters—the penalty coefficient (C) and the kernel parameter ($\upgamma$)—are considered. $\upgamma$ defines the influence of a single training example. The larger $\upgamma$ is, the closer other examples must be to be affected. C weighs the misclassified sample against the simplicity of the interface. A low C value smoothed the interface, while a high C value ensured that all samples were correctly classified by increasing the number of degrees of freedom of the model to select more support vectors. Grid search algorithm, which is a typical parametric search method in LibSVM was used to determined the best parameter combination of C and $\upgamma$.

By analyzing the overall situation of the study area comprehensively, we divided the land cover types in this study into bare land, understory vegetation (including low vegetation, grassland, etc.), shadows, and damaged *Pinus tabulaeformis.* (There are few mildly damaged or healthy *Pinus tabulaeformis*, as the study area covers the severely damaged area of *Pinus tabulaeformis*.). The SVM classification experiments were performed on UAV-based hyperspectral images of the entire area using the G-RBF with different parameter settings. We took samples based on pixels, each sample represented one pixel. A total of 5000 sample points were selected, of which 1250 were selected for each land cover type. The training and verification samples were divided in a ratio of 3:1. Combined with the ground survey information, the method of visual interpretation was used to complete the sample selection.

The overall accuracy (OA), kappa coefficient (Kappa, which is a measure index of the classification accuracy, it calculated based on the confusion matrix. The higher the Kappa is, the higher classification accuracy is.), and classification accuracy of damaged *Pinus tabulaeformis* (CADP) were evaluated to determine the optimal parameter settings for subsequent analysis. The pixel-wise classification map of each land cover type was represented as its corresponding probability map, and this map was regarded as the initial probability map of the corresponding land cover type.

#### Optimization of Initial probability maps by edge-preserving filter

The initial probability maps, which were constructed by the SVM classifier, do not incorporate any spatial information. Therefore, the probability maps appear noisy and are not aligned with actual object boundaries. Incorporating the characteristics of Chinese pine and damaged tree crowns, two edge-preserving filter (EPF) algorithms—the joint bilateral filter (JBF) and the guided filter (GF)—were selected to solve the edge structure problem.

##### Joint bilateral filter

The joint bilateral filter (JBF) is a processing method that removes internal noise by comprehensively analyzing the image spatial domain information and grayscale similarity to maintain the image edge information [46]. This method uses two Gaussian kernel functions that represent spatial distance (${G}_{{\sigma }_{d}}$) and range distance (${G}_{{\sigma }_{r}}$) as follows:

3 $${G}_{{\sigma }_{d}}=exp(-\frac{\left(\Vert i-j\Vert \right)}{{\sigma }_{d}^{2}})$$

4 $${G}_{{\sigma }_{r}}=\mathrm{exp}(-\frac{{\left|{I}_{i}-{I}_{j}\right|}^{2}}{{\sigma }_{r}^{2}})$$

Then, the weight of the bilateral filter is expressed as

5 $${W}_{i,j}=\frac{1}{{K}_{i}}exp(-\frac{\left(\Vert i-j\Vert \right)}{{\sigma }_{d}^{2}})\bullet exp(-\frac{{\left|{I}_{i}-{I}_{j}\right|}^{2}}{{\sigma }_{r}^{2}})$$

where *W* based on the input image represents the weight, *i* and *j* represent the pixel indices, *K* represents the normalized constant, *I* is the gray value of the pixel, ${\sigma }_{d}$ controls the size of the local window used to filter a pixel, and ${\sigma }_{r}$ represents the proportion of pixel value weight reduction in the local window.

For the JBF, the weight *W* depends on a new image instead of the original input image, which can be either a grayscale image or an RGB image and is called the guidance image. Further, *I* represents the guidance image, while *P* and *Q* represent the input image and filter output image, respectively. The JBF can be expressed as.

6 $${Q}_{i}=\sum_{j}{W}_{i,j}\left(I\right){P}_{j}=\sum_{j}\frac{1}{{K}_{i}}\mathrm{exp}(-\frac{\left(\Vert i-j\Vert \right)}{{\sigma }_{d}^{2}})\bullet \mathrm{exp}(-\frac{{\left|{I}_{i}-{I}_{j}\right|}^{2}}{{\sigma }_{r}^{2}})\bullet {P}_{j}$$

Based on (6), if the pixel $i$ and neighboring pixel $j$ have similar intensities or colors in the guidance image, and pixel j is close to pixel $i$, then the weight of pixel $j$ will be large. On the contrary, if the neighboring pixels have quite different intensities in the guidance image, the weight will be small.

##### Guided filter

The guided filter (GF) defines an output image as a model with a local linear correlation with the guidance image, which effectively smooths background details and preserves edge variations in the scene [47]. Further, *P,* $Q$ and $I$ can represent the input image, output image and guidance image, respectively. Within the local window $\omega$ of size $(2r+1)\times (2r+1)$ the local linear model of the guidance image can be expressed as.

7 $${Q}_{k}={a}_{k}{I}_{i}+{b}_{k}, \forall i\in {\omega }_{k}$$

$r$ represents the window radius, $ak$ and $bk$ are two parameters of the local linear model, and their values in different windows $\omega k$ are also different. Considering the purpose of the entire filter, the least-squares principle is used to perform regression fitting of the local linear model, and the cost function is set as follows:

8 $$E\left({a}_{k}, {b}_{k}\right)=\sum_{i\in {\omega }_{k}}\left({\left({a}_{k}{I}_{i}+{b}_{k}-{P}_{i}\right)}^{2}+\epsilon {a}_{k}^{2}\right)$$

where $\epsilon$ controls gradient changes. It is a regularization parameter for determining the degree of blurring for GF. GF can also be expressed in the following form:

9 $${Q}_{i}=\sum_{j}{W}_{i,j}\left(I\right){P}_{j}=\sum_{j}\frac{1}{{\left|\omega \right|}^{2}}\sum_{k:(i,j)\in {\omega }_{k}}\left(1+\frac{\left({I}_{i}-{\mu }_{k}\right)\left({I}_{j}-{\mu }_{k}\right)}{{\sigma }_{k}^{2}+\epsilon }\right)\bullet {P}_{j}$$

It can be understood that minimizing the cost function limits overall structural characteristics of the output image, and the local linear relationship enables the filtered image to replicate changes in the details of the guidance image as much as possible. That is the key to GF. In the GF, as the running time is not related to the size of the window, compared with other algorithms, GF can be processed with a larger window without affecting the computational efficiency.

##### Guidance image

In addition to the filter, another important factor affecting the filter results is the guidance image. The guidance image can provide and guarantee good edge structure information of the classifying feature to some extent. In general, the definition of an RGB image is slightly higher than that of a hyperspectral image when the hyperspectral sensor and high-definition digital camera installed on the UAV acquire data synchronously. This difference is due to the different field-of views of the two cameras. Although the difference is usually not very large, it cannot be ignored when extracting the land cover type, which contains prominent edge information, especially in the case of conifers or shrubs. Furthermore, the difficulty in data storage and analysis in the case of RGB images is much lower than that of hyperspectral images. Thus, we propose two methods for acquiring guidance images:

1. Principal component analysis (PCA) false color image: PCA of the preprocessed hyperspectral image using the false color image obtained by the combination of the first three principal components as the guidance image;
2. RGB image: The high-definition RGB image acquired synchronously with the corresponding hyperspectral image is used as the guidance image; spatial resampling is required before filtering because the spatial resolution of high-definition RGB images is different from that of hyperspectral data.

##### Parameter settings and accuracy evaluation of two EPFs

In this study, we set the parameters of the filters separately for the two types of guidance images, and we evaluated the optimization parameters. For the two EPFs, we set the filter size (${\sigma }_{d}$ and $r$) and the ambiguity (${\sigma }_{r}$ and $\epsilon$). The same as the parameter optimization of G-BRF, algorithm which similar to grid search is used in determining both two EPFs’ parameter combination.

To study the edge-preserving effect, the optimized probability map is evaluated by the mean structure similarity index (MSSIM) [48]. If the guidance image and filtered output image are represented by I and Q, respectively,

10 $$MSSIM(I,Q)=\frac{1}{M}\sum_{i=1}^{M}\frac{(2{\mu }_{I}{\mu }_{Q}+{c}_{1})(2{\sigma }_{IQ}+{c}_{2})}{({\mu }_{I}^{2}+{\mu }_{Q}^{2}+{c}_{1})({\sigma }_{I}^{2}+{\sigma }_{Q}^{2}+{c}_{2})}$$

where $M$ is the number of pixels in the image; ${\mu }_{I}$ and ${\mu }_{Q}$ are the mean values of the guided image and output image, respectively; ${\sigma }_{I}^{2}$ and ${\sigma }_{Q}^{2}$ are the variances of the guidance image and output image, respectively; and ${\sigma }_{IQ}$ represents the covariance of the guidance image and the output image, respectively. Further, ${c}_{1}={\left({K}_{1}L\right)}^{2}$,${c}_{2}={\left({K}_{2}L\right)}^{2}$, $L$ is the dynamic range of the pixel values, ${K}_{1}=0.01$, and ${K}_{2}=0.03$ [35]. The higher the value of the MSSIM, the greater the similarity between the two images, i.e., the better the preservation of the output image by the guidance image.

### Extraction of tree crowns damaged by *D. tabulaeformis*

By analyzing the classification effect of SVM and two types of EPFs under different parameter settings, the spectral-spatial classification framework for UAV-based hyperspectral data in the study area was established. First, the category of each pixel was determined using the maximum probability criterion by analyzing the optimized probability map, and the final classification results were obtained. Second, the range corresponding to the damaged *Pinus tabulaeformis* category was extracted based on the final classification results, and the identification of damaged pines was completed. Third, the OA, Kappa, and CADP were used to evaluate the extraction accuracy of damaged *Pinus tabulaeformis*.

## Data Availability

The datasets generated and/or analysed during the current study are not publicly available due [the funded project is under development] but are available from the corresponding author on reasonable request.

## Abbreviations

UAV: Unmanned aerial vehicle
SVM: Support vector machine
EPF: Edge-preserving filter
G-RBF: The Gaussian radial basis kernel function
JBF: The joint bilateral filter
GF: The guided filter
OA: The overall accuracy
Kappa: Kappa coefficient
CADP: Classification accuracy of damaged Pinus tabulaeformis
PCA: Principal component analysis
MSSIM: The mean structure similarity index
